# Cancer classification using the Immunoscore: a worldwide task force

**DOI:** 10.1186/1479-5876-10-205

**Published:** 2012-10-03

**Authors:** Jérôme Galon, Franck Pagès, Francesco M Marincola, Helen K Angell, Magdalena Thurin, Alessandro Lugli, Inti Zlobec, Anne Berger, Carlo Bifulco, Gerardo Botti, Fabiana Tatangelo, Cedrik M Britten, Sebastian Kreiter, Lotfi Chouchane, Paolo Delrio, Hartmann Arndt, Martin Asslaber, Michele Maio, Giuseppe V Masucci, Martin Mihm, Fernando Vidal-Vanaclocha, James P Allison, Sacha Gnjatic, Leif Hakansson, Christoph Huber, Harpreet Singh-Jasuja, Christian Ottensmeier, Heinz Zwierzina, Luigi Laghi, Fabio Grizzi, Pamela S Ohashi, Patricia A Shaw, Blaise A Clarke, Bradly G Wouters, Yutaka Kawakami, Shoichi Hazama, Kiyotaka Okuno, Ena Wang, Jill O'Donnell-Tormey, Christine Lagorce, Graham Pawelec, Michael I Nishimura, Robert Hawkins, Réjean Lapointe, Andreas Lundqvist, Samir N Khleif, Shuji Ogino, Peter Gibbs, Paul Waring, Noriyuki Sato, Toshihiko Torigoe, Kyogo Itoh, Prabhu S Patel, Shilin N Shukla, Richard Palmqvist, Iris D Nagtegaal, Yili Wang, Corrado D'Arrigo, Scott Kopetz, Frank A Sinicrope, Giorgio Trinchieri, Thomas F Gajewski, Paolo A Ascierto, Bernard A Fox

**Affiliations:** 1 INSERM, U872, Laboratory of Integrative Cancer Immunology, Paris, F-75006, France; 2 Université Paris Descartes, Paris, France; 3 Centre de Recherche des Cordeliers, Université Pierre et Marie Curie Paris 6, Paris, France; 4 Assistance Publique-Hopitaux de Paris, HEGP, Paris, France; 5 Society for Immunotherapy of Cancer, Milwaukee, WI, USA; 6 Infectious Disease and Immunogenetics Section (IDIS), Clinical Center and trans-NIH Center for Human Immunology (CHI), National Institutes of Health, Bethesda, Maryland, USA; 7 Cancer Diagnosis Program, National Cancer Institute, National Institutes of Health, Rockville, Maryland, USA; 8 Institute of Pathology, University of Bern, Bern, 3010, Switzerland; 9 Department of Pathology, Providence Portland Medical Center, Portland, OR, USA; 10 Department of Pathology, Istituto Nazionale per lo Studio e la Cura dei Tumori "Fondazione G.Pascale", Naples, Italy; 11 TRON - Translational Oncology at the University Medical Center of the Johannes Gutenberg University Mainz, Mainz, Germany; 12 Weill Cornell Medical College, Doha, Qatar; 13 Colorectal Surgery Department, Istituto Nazionale per lo Studio e la Cura dei Tumori, "Fondazione G.Pascale", Naples, Italy; 14 Department of Pathology, University of Erlangen, Erlangen, Germany; 15 Institute of Pathology, Medical University of Graz, Graz, Austria; 16 Division of Medical Oncology and Immunotherapy, University Hospital of Siena, Istituto Toscano Tumori, Siena, Italy; 17 Department of Oncology-Pathology, Karolinska Institutet, Karolinska University, Stockholm, Sweden; 18 Harvard Medical School and Massachusetts General Hospital, Boston, MA, 02114-2696, USA; 19 CEU-San Pablo University School of Medicine and HM-Hospital of Madrid Scientific Foundation, Institute of Applied Molecular Medicine (IMMA), Madrid, Spain; 20 Ludwig Institute for Cancer Research, Memorial Sloan-Kettering Cancer Center, New York, NY, USA; 21 University of Lund, Lund, Sweden; 22 Immatics Biotechnologies GmbH, Tübingen, Germany; 23 Experimental Cancer Medicine Centre, University of Southampton Faculty of Medicine, Southampton, United Kingdom; 24 Department Haematology and Oncology, Innsbruck Medical University, Innsbruck, Austria; 25 Molecular Gastroenterology and Department of Gastroenterology, Humanitas Clinical and Research Center, Rozzano, Milan, Italy; 26 Ontario Cancer Institute and Campbell Family Institute for Cancer Research, Princess Margaret Hospital, University Health Network, Toronto, ON, Canada; 27 Departments of Laboratory Medicine, Pathobiology & Radiation Oncology, Ontario Cancer Institute/Princess Margaret Cancer Centre, Toronto, ON, Canada; 28 Division of Cellular Signaling, Institute for Advanced Medical Research, Keio University School of Medicine, Tokyo, Japan; 29 Department of Digestive Surgery and Surgical Oncology, Yamaguchi University, Graduate School of Medicine, Yamaguchi, Japan; 30 Department of Surgery, Kinki University, School of Medicine, Osaka-sayama, Japan; 31 Cancer Research Institute, New York, NY, USA; 32 Department of Pathology, Avicenne Hospital, AP-HP, Bobigny, France; 33 Center for Medical Research, University of Tuebingen, Tuebingen, Germany; 34 Oncology Institute, Loyola University Medical Center, Cardinal Bernardin Cancer Center, Maywood, IL, USA; 35 School of Cancer and Imaging Sciences, University of Manchester, Christie Hospital NHS Trust, Manchester, UK; 36 Research Center, University Hospital, Université de Montréal (CRCHUM), Montréal, Québec, Canada ; Institut du Cancer de Montréal, Montréal, Québec, Canada; 37 Karolinska Institutet Department of Oncology-Pathology, Stockholm, Sweden; 38 Georgia Health Sciences University Cancer Center, Augusta, GA, USA; 39 Department of Pathology, Brigham and Women's Hospital and Harvard Medical School, Boston, MA, USA; Department of Medical Oncology, Dana-Farber Cancer Institute, Boston, MA, USA; 40 Department of Medical Oncology, Royal Melbourne Hospital, Melbourne, Australia; 41 Department of Pathology, The University of Melbourne, Melbourne, Australia; 42 Department of Pathology, Sapporo Medical University School of Medicine, Sapporo, Japan; 43 Department of Immunology and Immunotherapy, Kurume University School of Medicine, Kurume, Japan; 44 The Gujarat Cancer & Research Institute, Asarwa, Ahmedabad, India; 45 Department of Medical Biosciences, Pathology, Umea University, Umea, Sweden; 46 Pathology Department, Radboud University Nijmegen Medical Center, Nijmegen, The Netherlands; 47 Institute for Cancer Research, Center of Translational medicine, Xi’an Jiaotong university, Xian, China; 48 Department of Histopathology, Dorset County Hospital, DCHFT, NHS, Dorchester, UK; 49 MD Anderson Cancer Center, Houston, TX, USA; 50 Mayo Clinic and Mayo College of Medicine, Rochester, MN, 55905, USA; 51 Cancer Inflammation Program, Center for Cancer Research, National Cancer Institute and Trans-NIH Center for Human Immunology (CHI), National Institutes of Health, Frederick and Bethesda, Maryland, USA; 52 University of Chicago, Chicago, IL, USA; 53 Medical Oncology and Innovative Therapies Unit, Istituto Nazionale per lo Studio e la Cura dei Tumori, "Fondazione G. Pascale", Napoli, Italy; 54 Fondazione Melanoma Onlus, Napoli, Italy; 55 Laboratory of Molecular and Tumor Immunology, Earle A. Chiles Research Institute, Robert W. Franz Cancer Center, Providence Portland Medical Center, Portland, OR, USA; 56 Department of Molecular Microbiology and Immunology, Oregon Health and Science University, Portland, OR, USA

## Abstract

Prediction of clinical outcome in cancer is usually achieved by histopathological evaluation of tissue samples obtained during surgical resection of the primary tumor. Traditional tumor staging (AJCC/UICC-TNM classification) summarizes data on tumor burden (T), presence of cancer cells in draining and regional lymph nodes (N) and evidence for metastases (M). However, it is now recognized that clinical outcome can significantly vary among patients within the same stage. The current classification provides limited prognostic information, and does not predict response to therapy. Recent literature has alluded to the importance of the host immune system in controlling tumor progression. Thus, evidence supports the notion to include immunological biomarkers, implemented as a tool for the prediction of prognosis and response to therapy. Accumulating data, collected from large cohorts of human cancers, has demonstrated the impact of immune-classification, which has a prognostic value that may add to the significance of the AJCC/UICC TNM-classification. It is therefore imperative to begin to incorporate the ‘Immunoscore’ into traditional classification, thus providing an essential prognostic and potentially predictive tool. Introduction of this parameter as a biomarker to classify cancers, as part of routine diagnostic and prognostic assessment of tumors, will facilitate clinical decision-making including rational stratification of patient treatment. Equally, the inherent complexity of quantitative immunohistochemistry, in conjunction with protocol variation across laboratories, analysis of different immune cell types, inconsistent region selection criteria, and variable ways to quantify immune infiltration, all underline the urgent requirement to reach assay harmonization. In an effort to promote the Immunoscore in routine clinical settings, an international task force was initiated. This review represents a follow-up of the announcement of this initiative, and of the J Transl Med. editorial from January 2012. Immunophenotyping of tumors may provide crucial novel prognostic information. The results of this international validation may result in the implementation of the Immunoscore as a new component for the classification of cancer, designated TNM-I (TNM-Immune).

## Background

Conventional clinical and pathological risk prediction in cancer patients is usually achieved by histopathological evaluation of tissue samples obtained during surgical removal of the primary tumor. The histopathological characteristics used can include: the size of the tumor; tissue integrity; atypical cell morphology; histological grade; aberrant expression of protein and genetic markers; evidence of malignant transformation, senescence and proliferation; characteristics of the invasive margin (IM); depth of invasion; and the extent of vascularization. In addition, histological or radiological analyzes of tumor-draining and regional lymph nodes, as well as of distant organs, are carried out looking to identify evidence of metastases. In accordance with this classification system, the evaluation of cancer progression is performed longitudinally and then applied to estimate patient prognosis. The parameters used to predict disease-free (DFS), disease-specific (DSS) and overall (OS) survival are taken from statistical analysis of patients with similar disease progression characteristics and corresponding clinical outcome. Tumor staging (AJCC/UICC-TNM classification) summarizes data on the extent of the tumor burden (T), presence of cancer cells in draining and regional lymph nodes (N) and evidence of metastases (M). This classification, based only on tumor invasion parameters, has been shown to be valuable in estimating the outcome of patients with a variety of cancers [1-3].

However, these traditional classification tools provide limited information in estimating patient post-operative outcome. It is well known that clinical outcome can significantly vary among patients within the same histological tumor stage [4]. In some patients, advanced stage cancer can remain stable for years, and although rare, partial or full regression of metastatic tumors can occur spontaneously [5]. In contrast, relapse, rapid tumor progression and patient death is associated with approximately 20-25% of TNM I/II stage patients, despite complete surgical resection and no evidence of residual tumor burden or distant metastasis [5].

The predictive accuracy of this traditional staging system relies on the assumption that tumor progression is largely a cell-autonomous process. The focus of this classification is solely on the tumor cells and fails to consider and incorporate the effects of the host immune response [6]. Histopathological analysis of tumors has revealed the infiltration of inflammatory and lymphocytic cells [7]. Detailed intra-tumor analysis illustrates that these immune infiltrates are not randomly distributed. Tumor-infiltrating immune cells appear to be localized and organized within dense infiltrates in the center of the tumor (CT), at the IM of tumoral nests and in adjacent tertiary lymphoid structures (TLS). The presence of immune cells may reflect a distinct underlying biology of the tumor, as gene expression profiling and other assays have revealed the presence of a broad signature of inflammation. This signature includes evidence for innate immune activation, chemokines for innate and adaptive cell recruitment, immune effector molecules, and expression of immunoregulatory factors [8-10]. Immune infiltrates are heterogeneous between tumor types, and are diverse from patient to patient. All immune cell types may be found in a tumor, including macrophages, dendritic cells (DC), mast cells, natural killer (NK) cells, naïve and memory lymphocytes, B cells and T lymphocytes (which include various subsets of T cell: T_H_1, T_H_2, T_H_17, regulatory T cells (T_REGS_), T follicular helper cells (T_FH_) and cytotoxic T cells). The analysis of the location, density and functional orientation of different immune cell populations (termed the immune contexture [11,12]) in large collections of annotated human tumors has allowed the identification of components that are beneficial for patients and those that are deleterious [6,9,12-14]. Nonetheless, to implement any new tumor biomarker including immune infiltrates for routine clinical use, careful evaluation of its laboratory validity and clinical utility is essential [15].

Since tumor molecular features and immune reactions are inter-related, a comprehensive assessment of these factors is critical [16]. Examining the effects of tumor-host interactions on clinical outcome and prognosis clearly represents an evolving interdisciplinary field of molecular pathological epidemiology, the paradigm of which has recently been established [6,11,17,18]. Pathological immunity evaluation may provide novel information on prognosis and help identify patient cohorts more likely to benefit from immunotherapy.

### A new classification of cancer based on the tumor microenvironment

Increasing literature [9,11,13,14,19] and meeting reports [20-22] support the hypothesis that cancer development is influenced by the host immune system. A common theme has emerged, emphasizing the critical need to evaluate systemic and local immunological biomarkers. It is in agreement that this may offer powerful prognostic information and facilitate clinical decision-making regarding the need for systemic therapy [6,23]. Numerous data collected from large cohorts of human cancers (with sample sizes n = 415, 599 and 602, [9,13,14], respectively) demonstrated that the number, type and location of tumor immune infiltrates in primary tumors, are prognostic for DFS and OS. Altogether these immune parameters are designated as the immune contexture [11,12]. Notably, two large studies (with sample sizes n = 843 and 768, [24,25], respectively) have shown that tumor immune infiltrate patterns and subsets in colorectal cancer are significant prognostic biomarkers, even after adjusting for stage, lymph node count, and well-established prognostic tumor molecular biomarkers including microsatellite instability (MSI), *BRAF* mutation, and LINE- hypomethylation.

A potential clinical translation of these observations is the establishment of an Immunoscore, based on the numeration of two lymphocyte populations (CD3/CD45RO, CD3/CD8 or CD8/CD45RO), both in the CT and in the IM of tumors, as a clinically useful prognostic marker [14]. For instance, colorectal cancer (CRC) patients with local tumor, no detectable lymph node or distant metastasis are usually treated by surgery alone. However, 20-25% of these patients will have recurrence of their disease indicating that occult metastases were already present at the time of curative surgery. No tumor-associated marker predicts recurrence in these patients. The Immunoscore (“I”) utilizes the numeration of CD8 and CD45RO cells in the CT and the IM of resected tumors to provide a score ranging from Immunoscore 0 (“I”0), when low densities of both cell types are found in both regions, to Immunoscore 4 (“I”4), when high densities are found in both regions. This Immunoscore approach was applied to 2 large independent cohorts (n = 602). Only 4.8% of patients with a high “I”4, relapsed after 5 years and 86.2% were alive. In comparison, 72% of patients with a low score (“I”0 and “I”1) experience tumor recurrence and only 27.5% were alive at five years. These “I”0 and “I”1 patients potentially could have benefited from adjuvant therapy, had the Immunoscore been incorporated into the tumor staging [14].

The Immunoscore classification, demonstrating the prevalence of immune infiltrates, potentially has a prognostic significance superior to that of the AJCC/UICC TNM-classification system. For all patients with CRC stages I/II/III, multivariate Cox analysis revealed that the immune criteria remained highly significantly associated with prognosis. In contrast, the histopathologic staging system (T stage, N stage, and tumor differentiation) was no longer significant [13]. Tumor invasion was shown to be statistically dependent on the nature of the host-immune reaction. Indeed, the immune pattern remained the only significant criteria over the classical AJCC/UICC TNM-classification for DFS and OS, and led to an editorial entitled “TNM staging in colorectal cancer: T is for T cell and M is for memory” accompanying the publication by Mlecnik and Broussard et al. in the Journal of Clinical Oncology [13,26]. It has thus been suggested that the prevalence of post-surgical immune infiltrates, and not tumor status, is the key indicator for reoccurrence, metastasis and therefore clinical outcome.

These results suggest that once human cancer becomes clinically detectable, the adaptive immune response may play a critical role in preventing tumor recurrence. The ability of effector-memory T cells to recall previously encountered antigens leads to a protective response. Following primary exposure to antigen, memory T cells disseminate and are maintained for long periods of time [27]. The trafficking properties and the long-lasting antitumor capacity of memory T cells could result in long-term immunity in human cancer.

Although first described in CRC, the impact of the immune cytotoxic and memory T cell phenotype has been demonstrated in many other human tumors and appears to be a general phenomenon [23,28]. It is interesting to note that the implications of this immune phenotype apply not only to various organs of cancer origin (such as breast, colon, lung, head and neck, kidney, bladder, ovary, prostate), but also to various cancer cell types (adenocarcinoma, squamous cell carcinoma, large cell cancer, melanoma, etc).

A recent Nature Cancer Review meta-analysis [12] summarizes the impact of immune cells including B cells, NK cells, myeloid derived suppressor clls MDSC, macrophages, and all subsets of T cells on clinical outcome from more than 120 published articles. Beyond colorectal cancer, a strong T cell infiltration associated with good clinical outcome has been reported in many different tumours, including melanoma, head and neck, breast, bladder, urothelial, ovarian, esophageal, renal, prostatic, pancreatic, cervical, medulloblastoma, merkel cell carcinoma, hepatocellular, gastric, and lung cancers [12]. Thus, high densities of T cells (CD3+), of cytotoxic T cells (CD8+), and of memory T cells (CD45RO+) were clearly associated with a longer DFS (after surgical resection of the primary tumour) and/or OS.

The prognostic impact of other immune cells such as B cells, NK cells, MDSC, macrophages, and subset of T-helper populations, (T_H_2, T_H_17, T_REG_ cells) may differ depending on the type of cancer, and on the cancer stage [12]. In contrast, T cells, cytotoxic T cells, T_H_1 cells, and memory T cells were strongly associated with good clinical outcome for all cancer types [12]. Thus, general characteristics emerge in which cytotoxic T cells, memory T cells, and T_H_1 cells are associated with prolonged survival.

### The Immunoscore as a new approach for the classification of cancer

Considering the important role of the host immune signature in controlling tumor progression, it is now imperative to initiate the incorporation of the Immunoscore as a component of cancer classification [13,14] and a prognostic tool [23]. This strategy has a dual advantage: firstly, it appears to be the strongest prognostic factor for DFS and OS, particularly in early stage cancers and secondly, it could allude to potential targets for novel therapeutic approaches, including immunotherapy. Current immunohistochemical technologies allow the application of such analyses by laboratories concerned with routine diagnostic and prognostic assessment of tumors.

The inherent complexity of immunohistochemistry, in conjunction with protocol variability, analysis of different immune cell types, inconsistent tissue region selection criteria, combined with differences in conjunction with qualitative and semi-quantitative criteria to measure immune infiltration, all contribute to the variability of the results obtained, and raise the concern that specialized protocols and training may be required. It is therefore essential to pursue assay uniformity to reduce these limitations. Many markers, signatures, and methods have been described to evaluate the prognosis of cancer patients. Yet, very few such markers and laboratory assays are used in clinical practice. Thus, we believe that harmonization of an assay evaluating the “inflammation”, i.e. the Immunoscore of the tumor is essential. Analytical and clinical validation of the assay is required before the Immunoscore will reach clinical applicability for individual patients. However, current immunohistochemical technologies allow the application and cross-validation of such analysis in laboratories performing routine diagnostic and prognostic assessment of tumors. In order to be able to compare results in the future, and for the development of more effective prognostic and predictive markers to improve clinical decision-making, it is important to perform a standardized set of experiments. Assay harmonization should minimize data variability and allow worldwide correlations of Immunoscore results with clinical outcomes. Harmonization guidelines resulting from this process are expected to be simple to implement and will improve assay performance. Effective large-scale assay harmonization efforts have already been conducted for commonly used immunological assays of peripheral blood immune cell populations [29,30].

A fundamental parameter to determine the Immunoscore will include the immune cell density, calculated by numerical quantification of two lymphocyte populations, cytotoxic and memory T cells at the CT and the IM of tumors. This core criterion will establish prognosis of patient clinical outcome, regardless of the absence of other cancer associated prognostic markers, such as in early tumor stage (I/II) patients [14]. In human cancers, a high density of T_H_1/cytotoxic memory T lymphocytes, located both in the CT and IM of the primary tumor, is associated with long DFS and OS, in addition to low risk of relapse and metastasis. This was particularly illustrated in CRC [5,9,13,14,19], and should be applicable to most human tumors [23]. Thus, this Immunoscore classification may help identify the high-risk patients who would benefit the most from adjuvant therapy.

### Impact on response to cancer therapies

Whether the immune contexture of the primary tumor predicts therapeutic responses is of paramount importance for patient clinical management. Data based on immune signatures have established that a strong immune component is predictive of good response to chemotherapy in breast cancer [31-33], a tumor in which a high lymphocyte infiltrate is associated with higher response rate in neo-adjuvant therapy [34,35]. In hepatic metastases of CRC, high CD8 infiltrates in the IM predicts better response to chemotherapy and prolonged survival [36]. In melanoma, an immune signature displaying high expression of T_H_1 and cytotoxicity-associated genes, correlates with favorable clinical outcome to several different therapeutic vaccines [8]. In addition, high numbers of CD8 T cell infiltrates within metastatic melanoma correlated with prolonged survival [37]. However, the high T_H_1 and cytotoxic immune response associated with prolonged survival in patients receiving adjuvant therapies may not be a prediction of response to the therapy, but rather the fact that the host-immune response within the tumor protects the patient and prolongs patient life. To assess the impact of the Immunoscore as a predictive marker, it should be evaluated prospectively in randomized clinical trials.

### An open access call for a broad participation to the development of a task force dedicated to the evaluation of the Immunoscore in cancer patients

Over the past few years, the area of immune regulation at the level of the tumor microenvironment has gained a forefront position in cancer research, in CRC [9,12-14], in melanoma [38] and all other cancer types [6]. The Immunoscore was initially described several years ago [9], and more recently advances have been made in the development of the Immunoscore as a prognostic factor [13,14] that could be used in routine testing [39]. In an effort to promote the utilization of such Immunoscore in routine clinical settings worldwide, the Society for Immunotherapy of Cancer (SITC), the European Academy of Tumor Immunology (EATI), and “La Fondazione Melanoma Onlus”, initiated a task force on “Immunoscoring as a New Possible Approach for the Classification of Cancer” that took place in Naples, Italy, February 13^th^, 2012 [39]. This perspective represents a follow-up on this initiative, originally announced in a J Transl Med. editorial in January 2012 [39]. The working group, composed of international expert pathologists and immunologists, identified a strategy for the organization of worldwide participation by various groups for the validation of the Immunoscore. The objectives of the meeting included discussing: the role of immune system in cancer; a review of the AJCC/UICC-TNM classification of CRC; the role of the microenvironment in melanoma biology; the review of the AJCC classification of melanoma; the relevance of HLA-A2 in cancer prognosis and tumor malignancy; data utilizing the Immunoscore and a proposal for standardizing the operating procedures for the Immunoscore quantification. Furthermore, the international working group evaluated the feasibility of using the Immunoscore for the classification of cancer. Evidence-based selection of specific markers and their combinations for the Immunoscore was discussed including biological rationale, clinical use, synthetic meta-analysis of the Immunoscore, analytical performance, reagents availability and testing, metrics for decision making, cross-laboratory validation of methodology and identification of potential problems during development of other markers. Practical aspects of the validation of the assay by participating centers were proposed including consideration of cancer types, cancer stages, and the definition of a working group of pathologists for the validation phase.

**Figure 1:**
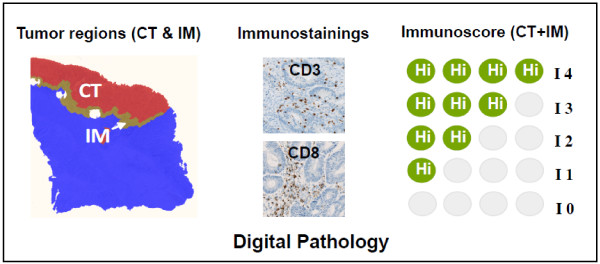
Immunoscore definition and method.

CRC has been most comprehensively studied and the prognostic significance of immunologic parameters has been best validated, thus special emphasis will be placed in this disease for this formal validation. As neo-adjuvant treatments are nowadays recommended for rectal cancer, it may be advisable to separate the validation of colon cancers and rectal cancers. Other cancer types, including melanoma and breast cancers were additionally discussed and their validation will follow. An independent international consensus panel of expert laboratories discussed cross-laboratory assay validation for the development of an Immunoscore prognostic method. As evaluation of cytotoxic memory CD8^+^ T cells (CD3^+^, CD8^+^, CD45RO^+^, Granzyme B^+^ (GZMB)) provides the best method to discriminate patient outcome, any combination of two of these aforementioned markers should have similar statistical power. Because of technical difficulties including background noise (CD45RO) and granular staining (GZMB), it was decided to employ the two easiest membrane stains, CD3 and CD8. Thus, the combination of two markers (CD3^+^ and CD8^+^) in two regions (CT and IM) was agreed for validation in standard clinical practice. Precise quantification will be performed on whole slide sections (Figure 1). For harmonization of the assay and reproducibility of the method, all laboratories agreed to test the prognostic value of specific immune cell infiltration following the recommended initial guidelines. The inherent complexity of quantitative immunohistochemistry underscored the urgent need to reach assay harmonization. The components of the Immunoscore are listed in Table 1. Additional markers could be added subsequently to refine the methodology even further if required. After worldwide validation, a consensus detailed protocol will be available.

**Table 1:** Current Immunoscore procedure and reagents

**Procedure**	**Current recommended steps**
Tumor selection	Block which is the most infiltrated by the immune cells and containing the core of the tumor (CT) and the invasive margin (IM)
Sample preparation	2 paraffin sections of 4-microns of the tumor block deposited in deionized water on Superfrost-plus slides
Immuno-histochemistry (IHC)	2 single stainings using IVD certified antibodies
Antigen retrieval	CC1 tris-based buffer pH8
Primary antibody	CD3 (2GV6, Ventana) and CD8 (C8/144, Dako)
Primary antibody diluant	K 004 (Clinisciences) for CD8
Secondary reagents	Ultraview TM DAB (Ventana)
Counterstaining	Hematoxillin II (Ventana)
Autostrainer	Benchmark XT (Ventana)
Scanner	NanoZoomer 2.0-HT (Hammamatsu)
Digital pathology	Architect XD software (Definiens)
Immunoscore quantification	Immunoscore Plug-in (INSERM / AP-HP)

To be used globally in a routine manner, evaluation of a novel marker should have the following characteristics: pathology-based, feasible in routine settings, simple, inexpensive, rapid, robust, reproducible, quantitative, standardized, and powerful. The Immunoscore fulfills all these keys aspects summarized in Table 2.

**Table 2:** Characteristics of a good marker and of the Immunoscore

**Must be**	**Immunoscore**	**Characteristics**
Routine	YES	Technic to be performed by pathologist using bright field and precise cell evaluation
Feasible	YES	Established pathology technics, using 2 regular whole slide FFPE section
Inexpensive	YES	Automatized immunohistochemistry
Rapid	YES	2 simple staining less costly than complicated molecular techniccs
Robust	YES	Autostainers, scanner, and digital pathology reduce the time to perform an Immunoscore
Reproducible	YES	Two strong membrane staining, with no background, allowing the numeration of individual cells
Quantitative	YES	Inter-observers variability is removed by the use of digital pathology, taking into account cell location and counts
Standardized	YES	Standardized operating procedure should be performed to insure reproducibility and worldwide comparisons
Pathology-base	YES	Necessity of pathologist expertise to validate cell type, cell location, and cell counts performed by digital pathology
Powerful	YES	The immunoscore has a prognostic value highly significant even in Cox multivariate including TNM classification^13^

The purpose of the Immunoscore worldwide task force is to validate these points.

The goals of the first ongoing initiative are the following:

1) to demonstrate the feasibility and reproducibility of the Immunoscore.

2) to validate the major prognostic power of the Immunoscore in routine settings for patients with colon cancer stage I/II/III.

3) to demonstrate the utility of the Immunoscore to predict stage II colon cancer patients with high risk of recurrence.

Thus, the benefit of the Immunoscore worldwide study would be to validate the feasibility, reproducibility, and prognostic value of the routine Immunoscore on colon cancer patients.

The goals of the next initiatives will be the following:

1) promote the worldwide use of the Immunoscore as a routine testing for cancer classification.

2) to validate the major prognostic power of the Immunoscore for patients with other cancer types (melanoma, breast, ovarian, endometrial, etc…).

3) to demonstrate the utility of the Immunoscore to predict response to treatments in clinical trials.

**Figure 2:**
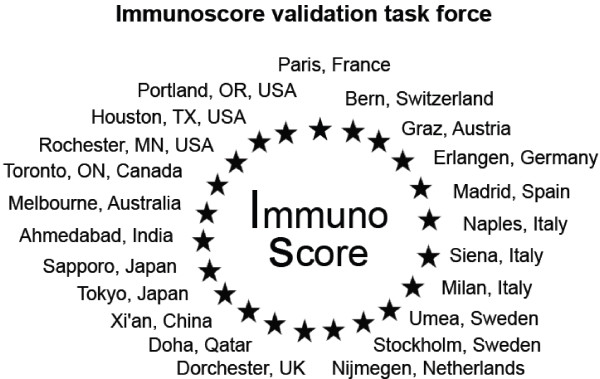
Worldwide expert centers participating in the Immunoscore task force.

In the inaugural World Immunotherapy Council meeting (February 21^st^ - 24^th^ 2012, Curacao), the Immunoscore task force, led by the Society for Immunotherapy of Cancer (SITC), received the support from several additional cancer immunology societies including; Biotherapy Development Association (BDA); Canadian Cancer Immunotherapy Consortium (CCIC); Cancer Immunotherapy Consortium (CIC) of the Cancer Research Institute (CRI); Association for Cancer Immunotherapy (CIMT); Committee for Tumor Immunology and Bio-therapy (TIBT); European Academy of Tumor Immunology (EATI); European Society for Cancer Immunology and Immunotherapy (ESCII); Italian Network for Tumor Biotherapy (NIBIT); Japanese Association of Cancer Immunology (JACI); Nordic Center for Development of Antitumor Vaccines (NCV-network); Progress in Vaccination Against Cancer (PIVAC); Adoptive engineered T cell Targeting to Activate Cancer Killing (ATTACK) and the Tumor Vaccine and Cell Therapy Working Group (TVACT). These groups share a clinical or basic interest in the immunobiology of the tumor microenvironment and will collaborate with worldwide expert pathologists to assess the validity of this new approach. Following the Immunoscore Workshop and the World Immunotherapy Council meeting, 22 international expert centers agreed to participate in this visionary enterprise. These participants represent 22 Centers Worldwide from 16 countries including Asia, India, Europe, North America, Australia, and Middle East (Figure 2). Additionally, pathologist associations and other medical specialty groups have been invited to participate.

A preliminary summary of this effort will be presented during the “Workshop on Tumor Microenvironment” prior to the SITC annual meeting (October 24^th^ - 25^th^ 2012, Maryland, USA). Finally a “Workshop on Immunoscore” (December 5^th^ 2012, Naples, Italy), will lead to the preparation of a summary document providing recommendations for the harmonization and implementation of the Immunoscore as a new component for the classification of cancer TNM-I (Immune).

## Conclusion

Prediction of clinical outcome in cancer is usually achieved by histopathological evaluation (AJCC/UICC-TNM classification) of tissue samples obtained during surgical resection of the primary tumor. However, it is now recognized that clinical outcome can significantly vary among patients within the same stage. The current classification provides limited prognostic information, and does not predict response to therapy. Recent literature demonstrated the importance of the host immune system in controlling tumor progression. Accumulating data, collected from large cohorts of human cancers, has demonstrated the impact of immune-classification, which has a prognostic value that may add to the significance of the current classification, and that has been demonstrated to be superior to the AJCC/UICC TNM-classification in colorectal cancer. It is therefore imperative to begin to incorporate the ‘Immunoscore’ into traditional classification, thus providing an essential prognostic and potentially predictive tool. Given the power of a proper immune evaluation of cancer patients, the Immunoscore is likely to be important for the field of cancer, beyond the field of tumor-immunology. In an effort to promote the Immunoscore in routine clinical settings, an international task force was initiated. The results of this international validation may result in the implementation of the Immunoscore as a new component for the classification of cancer, designated TNM-I (TNM-Immune). It is hoped that this effort will better define the prognosis of cancer patients, better identify patients at high-risk of tumor recurrence, to improve the quality of life by predicting and stratifying patients who will benefit from adjuvant therapies and, ultimately, to help save the lives of patients with cancer.

## Competing interests

The authors declare that they have no competing interests.

## Authors’ contributions

JG is coordinating this Immunoscore initiative, conceived the study, and wrote the manuscript. JG, FP initiated the Immunoscore project. FP, CL, AB, JG performed the initial experiments related to the Immunoscore. HKA participated in the drafting of the manuscript. FMM, TAG, BAF, JG from the SITC, initiated a task force and organized meetings on Immunoscore. PAA, from La Fondazione Melanoma Onlus organized initial meetings on Immunoscore. AL, CB, GB, FT, PD, AH, MA, LL, MM, FG, FP, FMM, BAF, JG were experts involved in the design of the immunoscore study, and expert pathologists participating to the inaugural Immunoscore workshop. MT, JPA, SO, GT, with their expertise, supported the Immunoscore initiative. GVM, SG, LH, CH, HSJ, CO, HZ, PSO, JODT, GP, MIN, RH, RL, AL, SNK, TF, BAF, JG, were experts participating to the WIC meeting and supporting the Immunoscore initiative. FP, AL, IZ, AB, CB, GB, FT, LC, PD, AH, MA, MM, FVV, LL, FG, PSO, PAS, BAC, BGW, YK, SH, CL, PG, PW, NS, TT, KI, RP, IDN, YW, CDA, SK, FAS, PAA, BAF, JG are expert participants of the initial worldwide Immunoscore task force study. All authors read and approved the final manuscript.
